# Multisensory visual-vestibular training improves visual heading estimation in younger and older adults

**DOI:** 10.3389/fnagi.2022.816512

**Published:** 2022-08-25

**Authors:** Grace A. Gabriel, Laurence R. Harris, Denise Y. P. Henriques, Maryam Pandi, Jennifer L. Campos

**Affiliations:** ^1^KITE-Toronto Rehabilitation Institute, University Health Network, Toronto, ON, Canada; ^2^Department of Psychology, University of Toronto, Toronto, ON, Canada; ^3^Department of Psychology, York University, Toronto, ON, Canada; ^4^Centre for Vision Research, York University, Toronto, ON, Canada; ^5^Department of Kinesiology, York University, Toronto, ON, Canada

**Keywords:** heading estimation, postural control, straight-ahead perception, training, aging, bimodal perception, self-motion, multisensory integration (MSI)

## Abstract

Self-motion perception (e.g., when walking/driving) relies on the integration of multiple sensory cues including visual, vestibular, and proprioceptive signals. Changes in the efficacy of multisensory integration have been observed in older adults (OA), which can sometimes lead to errors in perceptual judgments and have been associated with functional declines such as increased falls risk. The objectives of this study were to determine whether passive, visual-vestibular self-motion heading perception could be improved by providing feedback during multisensory training, and whether training-related effects might be more apparent in OAs vs. younger adults (YA). We also investigated the extent to which training might transfer to improved standing-balance. OAs and YAs were passively translated and asked to judge their direction of heading relative to straight-ahead (left/right). Each participant completed three conditions: (1) vestibular-only (passive physical motion in the dark), (2) visual-only (cloud-of-dots display), and (3) bimodal (congruent vestibular and visual stimulation). Measures of heading precision and bias were obtained for each condition. Over the course of 3 days, participants were asked to make bimodal heading judgments and were provided with feedback (“correct”/“incorrect”) on 900 training trials. Post-training, participants’ biases, and precision in all three sensory conditions (vestibular, visual, bimodal), and their standing-balance performance, were assessed. Results demonstrated improved overall precision (i.e., reduced JNDs) in heading perception after training. Pre- vs. post-training difference scores showed that improvements in JNDs were only found in the visual-only condition. Particularly notable is that 27% of OAs initially could not discriminate their heading at all in the visual-only condition pre-training, but subsequently obtained thresholds in the visual-only condition post-training that were similar to those of the other participants. While OAs seemed to show optimal integration pre- and post-training (i.e., did not show significant differences between predicted and observed JNDs), YAs only showed optimal integration post-training. There were no significant effects of training for bimodal or vestibular-only heading estimates, nor standing-balance performance. These results indicate that it may be possible to improve unimodal (visual) heading perception using a multisensory (visual-vestibular) training paradigm. The results may also help to inform interventions targeting tasks for which effective self-motion perception is important.

## Introduction

Accurately and precisely perceiving our own movements through space is important for safely navigating our environment. During tasks such as walking, driving, and standing, we receive dynamic information from several different sensory systems that our brains must quickly and efficiently integrate to coherently perceive self-motion. In real-world environments these individual sensory inputs are rarely experienced in isolation and integrating them typically improves perceptual precision (Meredith and Stein, 1986; Ernst and Banks, 2002; Angelaki et al., 2009; Fetsch et al., 2009; Butler et al., 2010, 2015; Gu et al., 2013). The greatest benefits of multisensory integration are often observed when sensory estimates are less reliable (The Principle of Inverse Effectiveness; Meredith and Stein, 1986), and older age is often associated with sensory decline. This suggests that older adults may particularly benefit from multisensory stimulation.

Two of the most important cues to self-motion perception are visual cues (e.g., optic flow; Gibson, 1950) and vestibular cues (Angelaki and Cullen, 2008). In younger and older adults, visual and vestibular cues are often weighted and integrated in an optimal manner that minimizes variability to inform self-motion perception (Fetsch et al., 2009, 2010; Butler et al., 2010, 2015; Angelaki et al., 2011; Karmali et al., 2014; Greenlee et al., 2016; Ramkhalawansingh et al., 2017, 2018). When visual and vestibular cues are congruent and redundant (as is typically the case during most everyday experiences), integrating these inputs improves the precision of perceptual estimates (Ernst and Bülthoff, 2004; Fetsch et al., 2010; Butler et al., 2015).

Aging is associated with changes in individual sensory functioning, such as vestibular perception (Roditi and Crane, 2012; Bermúdez Rey et al., 2016; Karmali et al., 2017; Beylergil et al., 2019; Kobel et al., 2021; Gabriel et al., 2022a) and visual perception (Owsley, 2011). In terms of self-motion perception specifically, relative to younger adults, healthy older adults are worse at perceiving the direction of visual motion (Bennett et al., 2007) and self-motion perception in particular (e.g., egomotion simulated with an optic flow field) (Warren et al., 1989; Mapstone et al., 2006; Snowden and Kavanagh, 2006; Billino et al., 2008; Duffy, 2009; Mapstone and Duffy, 2010; Kavcic et al., 2011; Velarde et al., 2013; Lich and Bremmer, 2014; Ramkhalawansingh et al., 2017, 2018), with some evidence indicating that a subset of healthy older adults are completely unable to estimate their heading direction using optic flow alone (Warren et al., 1989; Ramkhalawansingh et al., 2018). With regards to vestibular self-motion perception (passive movements in the dark), older adults demonstrate larger perceptual detection and discrimination thresholds relative to younger adults across most axes and directions (Roditi and Crane, 2012; Bermúdez Rey et al., 2016; Karmali et al., 2017, 2018; Beylergil et al., 2019; Gabriel et al., 2022a), except for in the yaw axis (Chang et al., 2014; Roditi and Crane, 2012; but see Bermúdez Rey et al., 2016). They do not, however, show differences relative to younger adults in discriminating forward linear heading direction using only vestibular inputs (Ramkhalawansingh et al., 2018). These results suggest that while certain aspects of vestibular perception decline with older age, other aspects may not (e.g., heading discrimination).

In addition to these unimodal changes, aging is also associated with changes in multisensory integration, which may become heightened with older age (Laurienti et al., 2006; Mahoney et al., 2011; Mozolic et al., 2012; Diaconescu et al., 2013; Freiherr et al., 2013; McGovern et al., 2014; de Dieuleveult et al., 2017), including heightened visual-vestibular integration (Ramkhalawansingh et al., 2017, 2018; Nestmann et al., 2020; Kenney et al., 2021). In other words, when multiple sensory inputs are congruent and redundant, older adults may experience greater perceptual benefits from integrating these sensory inputs compared to younger adults (Hughes et al., 1994; Cienkowski and Carney, 2002; Laurienti et al., 2006; Peiffer et al., 2007; Tye-Murray et al., 2010; Mozolic et al., 2011; McGovern et al., 2014; Ramkhalawansingh et al., 2016, 2018; de Dieuleveult et al., 2017). However, heightened integration can also lead to performance decrements when sensory inputs are in conflict. For instance, older adults are more susceptible to integrating incongruent sensory cues (e.g., visual and vestibular heading directions that differ) than younger adults, and they weight the less reliable sensory cue higher than is optimal (Ramkhalawansingh et al., 2018; Nestmann et al., 2020). These age-related changes in unisensory and multisensory processes may partially explain why older adults are particularly vulnerable to injury during tasks requiring accurate self-motion perception, such as when driving and walking (Public Health Agency of Canada, 2014; Center for Disease Control, 2018a,b). Therefore, improving self-motion perception might help protect against adverse outcomes, such as injuries due to falls or collisions, in the older adult population.

Very few studies have investigated whether heading perception (i.e., self-motion perception) can be improved through training. Recently, Klaus et al. (2020). found that younger adults’ perception of self-motion in the dark improved following training (i.e., feedback) when the trained motion type was a combination of roll and tilt (i.e., stimulating both the vestibular semicircular canals and otoliths simultaneously). However, Hartmann et al. (2013). showed that training was not effective when participants were moved exclusively in yaw (rotation around an Earth-vertical axis) or sway (linear motion from side to side) stimulating only the canals or otoliths, respectively. They also found that training was effective in improving sway (but not yaw) motion perception if vision was also provided during training (i.e., if training was multisensory).

With regards to visual-only training, Kuang et al. (2020) showed that younger adults can improve their visual estimates of left/right heading direction from optic flow fields through feedback-based training, and Gibson et al. (2020) also showed that training could improve vertical heading accuracy (e.g., down-and-forward vs. down-and-backward). But no studies have yet evaluated whether vestibular and/or visual self-motion training can improve heading perception in older adults. This present study therefore investigates the effects of multisensory training on heading perception [biases and just-noticeable differences (JNDs)] and assesses whether any benefits of perceptual training might transfer to other performance-related domains such as improving standing-balance. We also examine whether potential training-related improvements may be more apparent in older relative to younger adults.

Here, we trained older and younger adults in a passive visual-vestibular heading discrimination task (forward left/right judgments). Participants’ heading estimation biases and JNDs were measured both pre- and post-training for three different sensory conditions: (1) visual-only (they were visually moved through a virtual starfield using a head-mounted display), (2) vestibular-only (they were physically moved on a six-degree-of-freedom motion platform in the dark), and (3) bimodal (visual and vestibular cues combined). We also assessed potential far-transfer-of-training effects by collecting pre- and post-training posturography measures during a quiet standing balance task under full and reduced sensory conditions.

## Materials and methods

### Participants

Participants were screened over the phone and were invited to participate only if they reported no history of stroke, seizures, diagnosed vestibular disorder, disabling musculoskeletal disorder, acute psychiatric disorder, eye disease (e.g., glaucoma or cataracts), diagnosed mild cognitive impairment, dementia, or hearing loss. Ultimately, 14 older adults and 13 younger adults met the screening-eligibility criteria and were invited to participate in the study. The sample size was based on (and/or exceeded) previous visual-vestibular training studies (Hartmann et al., 2013). Older adult participants completed an in-lab baseline assessment session (see below), which consisted of a battery of sensory, cognitive, and mobility tests, a sub-set of which were used to ensure that certain eligibility criteria were met (visual acuity, pure tone audiometry, cognitive impairment). Data from three older adult participants were excluded due to an inability to understand task instructions (*n* = 1) or because they did not have their prescription glasses for both pre-training and post-training sessions (*n* = 2). Data from two younger adults were also excluded due to not completing the post-training session. Thus, the data from 11 older adults (*M*_*age*_ = 71.54 years, *SD* = 6.70, females = 9, males = 2) and 11 younger adults (*M*_*age*_ = 23.73 years, *SD* = 5.18, females = 8, males = 3) were included in the analyses. Participants provided written informed consent and were compensated $20/h for their participation. This study was approved by the Research Ethics Boards of the University Health Network (Protocol Number: 18-5331.0) and the University of Toronto (Protocol number: 00037394).

### Baseline assessment session

Older adult participants completed a series of sensory, cognitive, and mobility assessments. Both age groups completed the visual assessments. If participants wore corrective lenses during the experimental procedure, they were required to wear those same corrective lenses during the baseline assessment testing. Results of these assessments are given in Table 1.

**TABLE 1:** Summary of baseline assessment measures.

	Older adults	Younger adults	*P*-value
Age	71.55 (6.70)	23.72 (5.18)	<0.001
Sex (*n*) (Female : Male)	9:2	8:3	–
**ETDRS^a^ (logMAR)**			
Right	0.13 (0.06)	0.07 (0.15)	0.36
Left	0.21 (0.18)	0.10 (0.20)	0.26
**Pelli-Robson (log-CS)**			
Right	1.50 (0.14)	1.78 (0.13)	<0.001
Left	1.54 (0.13)	1.82 (0.20)	<0.001
Binocular	1.63 (0.05)	1.95 (0.09)	<0.001
**Randot stereo test (Arcsec)**			
Circles	36.82 (15.70)	30 (7.75)	0.22
Forms	340.91 (126.13)	250 (0)	0.04
Animals	140.91 (70.06)	113.64 (25.23)	0.29
PTA^b^	21.00 (14.43)	–	–
Speech Spatial, spatial and qualities hearing scale	7.76 (1.28)	–	–
MoCA^c^	28.30 (1.95)	–	–
TUG^d^	8.71 (1.42)	–	–

p-values represent the results of independent samples, two-tailed t-tests between the two groups.

With the exception of “Sex” which is reported as sample size (n), all scores and values are reported as averages with standard deviations in parentheses.

^*a*^ETDRS = Early Treatment Diabetic Retinopathy Study visual acuity test.

^*b*^PTA = Binaural Pure Tone Average; frequencies tested: 500, 1,000, 2,000, and 4,000 Hz, inclusive.

^*c*^MoCA, Montreal Cognitive Assessment.

^*d*^TUG, Timed-Up and Go Task.

#### Vision screening

##### Visual acuity

To measure visual acuity, participants stood 4 m away from an ETDRS (Early Treatment Diabetic Retinopathy Study Research Group, 1985) visual acuity chart and were asked to read the letters on the chart. They were instructed to read the chart from left-to-right for their left eye, and right-to-left for their right eye, beginning with their better-seeing eye. When participants could not read a letter, they were asked to guess. Testing stopped once the participant made three errors on one line. For each eye, the last line read with at least three correct letters was recorded and later converted into a LogMAR score. All participants had a LogMAR score which was less (i.e., better) than 0.5 (see Table 1), indicating visual acuity in the normal or near-normal range (Colenbrander, 2002, 2010).

##### Pelli-robson contrast sensitivity

To measure contrast sensitivity, participants stood 1 m from a Pelli-Robson contrast sensitivity chart (Pelli et al., 1988) and were instructed to read the letters from left-to-right for each eye, beginning with their better-seeing eye and then with both eyes. Testing continued until participants reported two out of three letters in a triplet incorrectly. Participants’ log contrast sensitivity score was recorded as the last triplet for which they had correctly read at least two out of the three letters (Table 1), and all participants obtained scores within the range of normal (or better) for their age-group (Mäntyjärvi and Laitinen, 2001).

##### Randot stereo test

The Randot Stereo test (12%; Stereo Optical Company)^1^ was used to assess stereovision (Table 1). The test booklet was held by the experimenter 16 inches from the participant. Participants were instructed to wear polarizing viewers (over their prescription glasses, if necessary) and report the forms or images displayed in the booklet. Seconds of arc at 16 inches were recorded for each subtest.

#### Auditory screening

##### Pure-tone audiometry

Given that declines in vestibular functioning may be associated with age-related hearing loss (Viljanen et al., 2009; Lin and Ferrucci, 2012; Zuniga et al., 2012; Campos et al., 2018; Carpenter and Campos, 2020; Lubetzky et al., 2020; Gabriel et al., 2022b) all older adult participants were screened to ensure normal hearing. Audiometric testing was completed as per the guidelines established by the International Organization of Standardization (ISO; ISO 8253-1, 1989). Pure-tone audiometry was used to determine audiometric hearing thresholds using a Grason-Stadler 61 Clinical Audiometer (GSI-61; Grason-Stadler Inc., Eden Prairie, MN) and Telephonics TDH-50P headphones (Telephonics Corporation, Farmingdale, NY). Testing was performed in a double-walled, sound-attenuating booth (Industrial Acoustics Company, Inc., New York, NY). Frequencies tested were from 250 to 8,000 Hz, inclusive. Binaural pure-tone audiometric (PTA) thresholds were averaged across 500, 1,000, 2,000, and 4,000 Hz (Table 1). All but *n* = 3 older adult participants had an average binaural (and better ear) PTA average below the 25 dB HL cut-off for hearing loss (World Health Organization, 1991).

##### Speech, spatial and qualities of hearing scale

The Speech, Spatial and Qualities of Hearing Scale comprises three separate scales that measure subjective abilities to hear spoken language in day-to-day settings (“Speech”), to accurately perceive the direction or location of a sound source (“Spatial”), and to perceive the clarity of a given real-world auditory stimulus (“Qualities”) (Gatehouse and Noble, 2004). The maximum average test score is 10 points, which is the total combined average of all tested items and indicates that the participant reported no hearing difficulties. All but 2 older adults completed this assessment (Table 1).

#### Cognition

Mild cognitive impairment was screened for using the Montreal Cognitive Assessment (MoCA; Nasreddine et al., 2005). The test assesses general cognitive abilities by examining several domains of cognitive functioning including attention, executive function, memory, and language and is scored out of a total of 30 points. In this study, level-of-education adjusted scores are reported and all participants obtained a score of 26 or higher (the common cut-off for mild cognitive impairment).

#### Mobility

Walking, balance, and mobility impairments were assessed using the Timed-Up-and-Go (TUG) task. Four older adults did not complete this task. For each trial, participants were seated in a chair with armrests and instructed to stand and walk at a comfortable pace to a clearly delineated point 3 m away, turn around, and sit back down again. Participants completed this task twice while the experimenter timed each trial. The cut-off time for community-dwelling older adults who are not at risk of falling is 12s or less (Shumway-Cook et al., 2000; Bischoff et al., 2003). All older adults who were tested met this criterion.

### Experimental sessions

**FIGURE 1:**
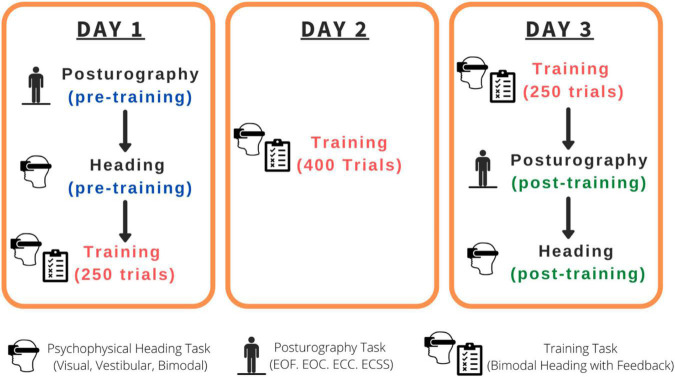
Order of the tasks completed for each participant. EOF, Eyes-open on a firm surface; EOC, Eyes-open on a compliant surface; ECC, Eyes-closed on a compliant surface; ECSS, Eyes-closed on a compliant surface, wearing passive sound-suppressing headphones.

The combined experimental sessions for this study were roughly 7 h in duration per participant spread across three separate days within a 2-week span (see Figure 1): Day 1 (pre-training psychophysical heading judgments and posturography tasks followed by the first 250 training trials: 2.5 h), Day 2 (400 training trials: 2 h), Day 3 (250 training trials, post-training psychophysical heading judgments and posturography tasks: 2.5 h). The psychophysical tasks consisted of three conditions: visual-only, vestibular-only, visual-vestibular (combined and congruent; bimodal). Training trials were bimodal visual-vestibular trials with feedback provided.

#### Psychophysical heading judgment task

##### Stimuli and apparatus

###### Visual condition

**FIGURE 2:**
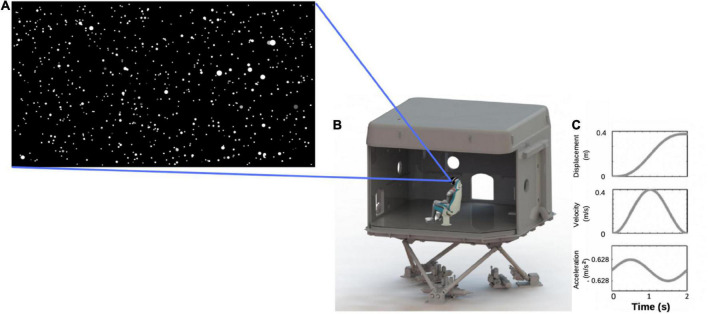
**(A)** Screen capture of the starfield viewed by participants; **(B)** illustration of a participant wearing a head-mounted display, seated in a chair located within the laboratory that was mounted on top of the 6-degree-of-freedom hydraulic hexapod motion platform; **(C)** motion profile for one trial.

Visual stimuli were rendered using the platform *Unity* version 2019.2.2f1 by Unity Technologies Inc. (Unity, 2019). The visual display consisted of a 120 × 120 × 50 m virtual space, presented through a stereoscopic head-mounted display (HMD; HTC Vive, 2016) whose AMOLED (active-matrix organic light-emitting diode) screen resolution was 1,080 × 1,200 pixels per eye, with a 90 Hz refresh rate, and 110° diagonal field of view. The virtual space was populated with 2,000 white spheres with a visual angle of 0.1° at its furthest distance (i.e., smallest size; Figure 2A). Forward visual self-motion was simulated by moving the spheres toward and past the viewer. The size of the spheres increased as their distance to the viewer decreased within the virtual space. The movement of the starfield followed a smooth sinusoidal acceleration and deceleration profile, beginning at 0 m/s and reaching a peak velocity of 0.4 m/s after 1 s with a peak acceleration of 0.628 m/s^2^. The motion then decelerated to 0 m/s for another 1 s (Figure 2C). As such, each trial lasted 2 s, allowing participants to visually travel 0.4 m through the virtual starfield. At the start of each condition, participants were moved toward a point 25° to the left or right of straight ahead. This heading angle then widened or narrowed as a function of an adaptive staircase, described in more detail below. Participants remained securely seated within the laboratory for the duration of the experiment and used a videogame controller to submit their direction-discrimination responses (“left” or “right”).

###### Vestibular condition

The experiment took place in one of the KITE-Research Institute’s 8 m^3^ fiberglass laboratories, which was mounted on a 6-degree-of-freedom hydraulic hexapod motion platform (Bosch-Rexroth HyMotion 11000; Figure 2B). Participants were seated on a specially constructed bucket-seat and secured with a four-point harness to reduce torso, head, and limb movement. The seat was cushioned to reduce vibrotactile cues to the body. Participants also rested their feet on a foam mat and wore an inflatable neck-pillow to further reduce movement of, and vibrotactile cues to, the neck and legs, thereby limiting the availability of extra-vestibular cues to motion. To reduce visual-input, and for consistency, participants continued to wear the head-mounted display that they wore during the visual-only condition, but the screen was dark (black) for the duration of each 2 s trial.

Motions were applied through movements of the motion platform, which used a smooth, sinusoidal acceleration and deceleration profile. The maximum acceleration and peak velocity were identical to those of the visual stimuli (i.e., ± 0.628 m/s^2^ and peak velocity of 0.4 m/s), meaning that the lab moved 0.4 m during every 2 s trial (see Figure 2C). This motion profile was similar to that used by Ramkhalawansingh et al. (2018) and is well above human acceleration detection thresholds. Again, this condition started with movement direction displaced 25° to the left or right of straight ahead, with the angle changing as a function of an adaptive staircase procedure throughout the trials, as described below.

###### Visual-vestibular (bimodal) condition

During the bimodal visual-vestibular condition, participants were presented with simultaneous, congruent visual and vestibular input, with the same motion profiles described above.

### Procedure

Every participant completed, (1) pre-training psychophysical tasks (visual-only, vestibular-only, bimodal) and pre-training posturography tasks, (2) training task, and (3) post-training posturography tasks and post-training psychophysical tasks (visual-only, vestibular-only, bimodal), across 3 days (see Figure 1 for a summary of the timeline).

#### Psychophysical heading task

**FIGURE 3:**
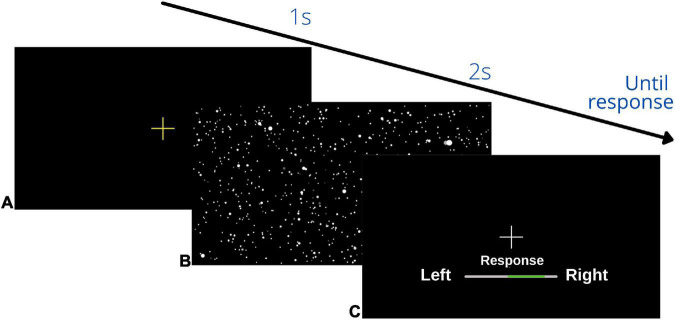
Schematic overview of visual display during the psychophysical heading task: **(A)** initial yellow fixation cross to signal the start of the trial, **(B)** heading movement: the starfield was present for the visual, bimodal and training trials, but during the vestibular condition the display was black, **(C)** response screen: participants pressed and held their response until the bar on the display was filled in green in the direction of their heading judgment (left/right) (2 s). This sequence repeated until the end of the block.

On Day 1 (pre-training) and Day 3 (post-training) we used two, randomly interleaved Parametric Estimation by Sequential Testing (PEST) staircases (Taylor and Creelman, 1967) for each of the three condition (visual-only, vestibular-only, and bimodal) separately to assess the bias and JND of participants’ heading percepts. Each trial began with a yellow fixation cross in the VR display, followed by a movement. After each movement, a white fixation cross was presented straight ahead of the participant and the participant was instructed to indicate whether they had been moved to the left or right of straight ahead. Participants responded using a videogame controller by pressing and holding the joystick to the left or right for 2 s. The 2 s period was indicated by a green bar that grew to full size in 2 s (see Figure 3).

**FIGURE 4:**
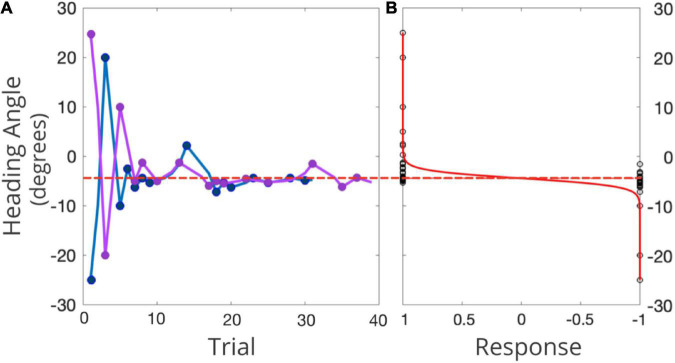
**(A)** Example of two interleaved PEST staircases for one representative participant in one condition. The right-starting PEST is plotted in pink and the left-starting PEST in blue. 0° represents straight-ahead, with positive values representing rightward angles, and negative values leftward angles. The red dashed line represents the participants’ bias as inferred from the logistic function. **(B)** Black circles represent the participant’s response (1 = right, –1 = left) for each presented heading angle, in the solid red line is the fitted psychometric function. The dotted red line represents the midpoint of this function (i.e., participant’s perception of straight ahead).

Conventional PEST rules were used to determine the next heading angle to be presented in each staircase (Taylor and Creelman, 1967; see Figure 4A for an example). The initial step size was 45° and the initial focus of expansion (FOE) of the headings were 25° to the left of straight ahead for one staircase, and 25° to the right of straight ahead for the other. The largest angle that could be presented was 50°. Each staircase terminated after 15 reversals. MATLAB was used to fit the data to a logistic function, where the 50% point represented participants’ perceptual bias—the heading where they were equally likely to choose left or right of straight ahead (Figure 4B). The slope of that function (defined as ±23.1% of the bias) was used to represent the JND of their heading judgments. Each condition took approximately 20 min to complete and the order in which the three conditions were tested was randomized across the participants.

#### Training task

During the second half of the Day 1 session, during the full session on Day 2, and during the first half of the Day 3 session (see Figure 1 for a timeline), participants completed 900 bimodal training trials total. During each training trial, participants were physically and visually moved (congruent and bimodal) in a direction either to the right or left of straight-ahead and asked to judge their heading direction relative to straight-ahead. Following their “left” or “right” responses they received feedback of either “Correct” in green, or “Incorrect” in red on the visual display. The heading angles were chosen randomly from a range centered around true straight-ahead (0°), ±67% of their angular bias from the pre-training bimodal psychophysical heading estimation task. Specifically, we took 67% of each participant’s bias and presented them with values chosen randomly from the range of plus and minus this value (e.g., a participant with a perceptual bias of +10 would be presented with heading angles between −6.7 and +6.7° from true straight-ahead). The training range was chosen to ensure that the deviations from true straight-ahead were not too easy (in which case there would be no added value from receiving feedback) or too difficult (i.e., imperceptible). Guidance for these values was also provided by the training range selected by Hartmann et al. (2013).

#### Posturography task

Immediately before the pre-training and post-training psychophysical heading task on Days 1 and 3, participants completed a posturography task. Participants were asked to stand on a force plate (AMTI MSA-6 MiniAmp strain gage amplifier) for 30 s (Scoppa et al., 2013). The center of pressure (COP) path length (cm), velocity (cm/s), and velocity root-mean-square (RMS; cm/s) were measured during quiet standing. Participants stood with feet parallel and wore a loose harness throughout the procedure to protect against a potential loss of balance. This posturography task was completed under four counterbalanced conditions. Participants either stood (1) directly on the forceplate with their eyes open on a firm surface (EOF; “firm surface”), (2) with their eyes open while standing on a piece of high-density, compliant foam placed directly on the forceplate (EOC; “compliant surface”; AIREX, Balance-Pad; 50 × 41 × 6 cm; density = 55 kg/m^2^), (3) with their eyes closed on the compliant surface (ECC), or (4) with their eyes closed on the compliant surface, while wearing sound-suppressing headphones (ECSS).

The forceplate data were collected at a sampling rate of 1,000 Hz. The first 5 s of the data were removed for each condition and the remaining data were passed through a 2nd order zero-lag dual-pass Butterworth filter with a 6 Hz cut-off frequency. MATLAB was used to extract mean COP path lengths, velocity, and velocity-RMS. Path length was defined as the absolute total length of sway in centimeters recorded in each condition, average velocity as the COP excursion divided by trial time, and velocity-RMS as the square-root of the mean of squares of the velocity measures. Longer path lengths, and higher velocities and velocity-RMS indicated more variable postural sway.

### Data analysis

All analyses were run using the biases and JNDs obtained as described above in R 3.6.0 (R Core Team, 2017). While the analyses presented below use raw, unwinsorized data, analyses with winsorized data can be found in Supplementary material 1. Two separate mixed-factorial ANOVAs, 2 (Age Group; younger, older) × 3 (Psychophysical Condition; visual-only, vestibular-only, bimodal) × 2 (Session; pre-training, post-training), were conducted to evaluate the extent to which participants’ perceptual biases and JNDs (dependent variables) changed in the older and younger groups following training, for each of the three psychophysical conditions (visual-only, vestibular-only, bimodal). Post-hoc *t*-tests were Tukey-corrected for multiple comparisons. We also examined the magnitude of change in biases and JNDs for these participants, which we calculated using difference scores pre- vs. post-training. Specifically, we took the absolute value obtained post-training and subtracted it from the absolute value obtained pre-training [Bias_*DifferenceScore*_ = *abs*(*Bias*_*Session*1_)−*abs*(*Bias*_*Session*3_); JND_*DifferenceScore*_ = *abs*(*JND*_*Session*1_)−*abs*(*JND*_*Session*3_)]. Positive scores indicated an improvement (biases or JNDs larger in Session 1 than in Session 3), while negative values indicate the opposite. We conducted two different 2 (Age Group) by 3 (Psychophysical Condition Difference Score) mixed-factorial ANOVAs with bias or precision as the dependent variable.

Importantly, three out of 11 older adults in this study were unable to complete the visual-only condition during the pre-training session. Specifically, the data obtained from the left- and right-starting PESTs in these three older adult participants for the visual-only condition did not converge due to essentially random responding (see Supplementary material 2 for their raw data). The experimenter re-explained the task to them numerous times to ensure that it was not a problem of task comprehension. They were also able to do the other two pre-training conditions (vestibular and bimodal). As such, we have omitted these participants’ heading data from the group analysis, and instead report their data separately.

We conducted pairwise *t*-tests to assess whether the difference scores, when collapsing across Age Groups, were significantly different from zero (i.e., no training effect). We also report the number of participants who demonstrated numeric post-training improvements, both including and excluding the three older adults who were unable to complete the visual-only condition pre-training (see Supplementary material 3).

Using a Maximum Likelihood Estimation (MLE) model we calculated the predicted optimal JNDs and biases for each condition and compared these predictions to the observed values using paired-sample *t*-tests.

For the training data, each of the 900 trials were coded as “1” for correct, and “0” for incorrect. This classification allowed us to calculate the number of correct responses obtained by participants within a given bin. Specifically, the percent correct for every 50 trials was calculated creating 18 bins of 50 trials where larger values indicated a greater percentage of correct responses than smaller values. Average of percent correct responses were computed for each of the 3 days separately (i.e., Day 1 was 250 trials, Day 2 was 400 trials, and Day 3 was 250 trials). A 2 (Age Group; younger, older) × 3 (Days; 1, 2, 3) mixed-factorial ANOVA was conducted to examine the extent to which performance changed over the course of training and whether there were any age-related differences.

With respect to the posturography measures, three mixed-factorial ANOVAs, 2 (Age Group; younger, older) × 4 (Posturography Condition; EOF, EOC, ECF, ECSS) × 2 (Session; pre-training, post-training) were conducted for COP (1) path length, (2) velocity, and (3) velocity-RMS. Post-hoc *t*-tests used were Tukey-corrected for multiple comparisons. Posturography data from three younger adults were excluded from all analyses due to a technical error during Session 1.

## Results

### Heading

#### Just-noticeable difference values

**FIGURE 5:**
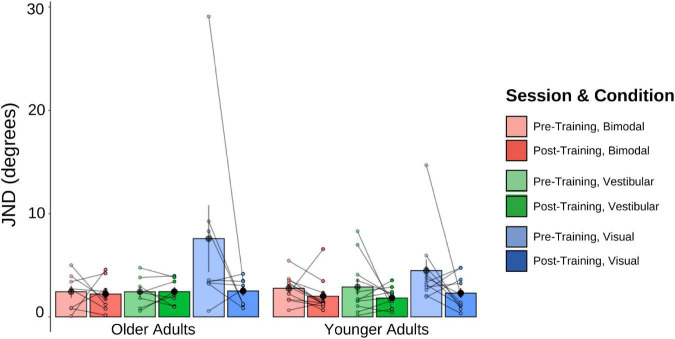
Mean and individual JND values plotted for each Age Group and sensory condition, pre-training (lighter shades) and post-training (darker shades). Individual data points are also plotted, with lines connecting each participant’s pre-training JND to their post-training JND for each of the three sensory conditions (visual-only, vestibular-only, bimodal). Black dots represent means, plotted with standard error bars.

The pre-and post-training JNDs are shown in Figure 5. The 2 (Age Group) × 3 (Psychophysical Condition) × 2 (Session) mixed-factorial ANOVA on JND values revealed a main effect of Session [*F*(1, 17) = 9.45, *p* = 0.007], indicating that pre-training JNDs were significantly larger (i.e., worse) than post-training JNDs. It also showed a main effect of Condition [*F*(1.48, 25.14) = 5.89, *p* = 0.013], with *post-hoc t*-tests revealing that JNDs in the vestibular-only condition were significantly smaller than the JNDs in the visual-only condition [*t*(17) = −2.72, *p* = 0.037], and results trending to suggest that JNDs in the bimodal condition were significantly smaller than those in the visual-only condition [*t*(17) = −2.54, *p* = 0.053] (Figure 5).

No other main effects or interactions were significant. This includes the Session × Condition interaction which was only trending, [*F*(1.45, 24.65) = 3.04, *p* = 0.080], with *post-hoc t-*tests showing this trend to be driven by the visual-only data, [*t*(17) = 2.56, *p* = 0.020].

#### Just-noticeable differences: Absolute difference scores

**FIGURE 6:**
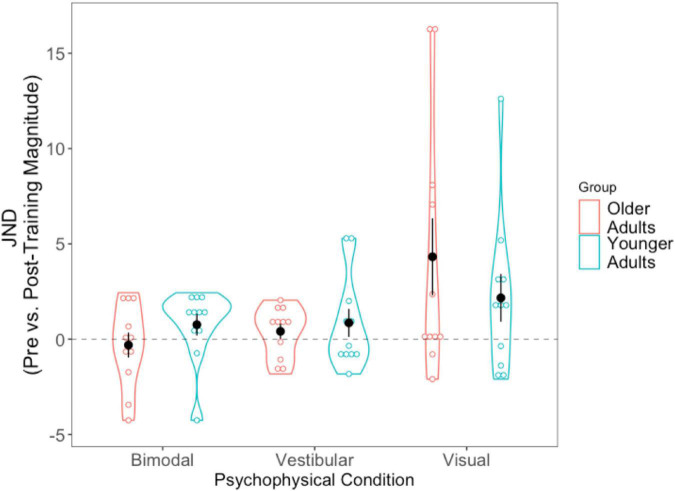
Difference scores for JNDs, across all three psychophysical conditions, for each of the two age groups. Black dots represent means, and error bars represent standard error. Individual data points are represented by the blue (younger adults) and red (older adults) circles. Positive values indicate improvement. Black dashed line represents “0” (i.e., no change after training).

For each of the conditions, we took the absolute value of the post-training JND and subtracted it from the absolute pre-training JND (i.e., Session 1 minus Session 3). Positive scores thus indicate improvement (JNDs being larger, or worse, in Session 1 than Session 3), while negative values indicate the opposite. A 2 (Age Group) × 3 (Condition Difference Score) mixed-factorial ANOVA showed a significant main effect of Condition, [*F*(1.36, 27.14) = 4.43, *p* = 0.034]. Tukey-corrected *post-hoc t*-tests showed the visual condition to be driving this effect—with larger difference scores (improvement) for the visual condition compared to the bimodal condition [*t*(40) = −2.74, *p* = 0.024], and trending significance for the vestibular condition [*t*(40) = −2.38, *p* = 0.057], indicating significantly greater precision for the visual condition following training (Figure 6).

We then conducted three separate pairwise *t*-tests to compare each condition’s JND difference scores (visual, vestibular, bimodal) with “0,” in order to assess whether the changes in JNDs following training differed significantly from zero. The results showed that the post-training reduction in JNDs in the visual condition was significantly different from 0, [*t*(21) = −2.728, *p* = 0.012] but the vestibular-only and bimodal conditions were not significantly different from 0 (*p* > 0.05).

We also tallied the number of participants who demonstrated numeric post-training improvements (lower JND) for each of the three sensory conditions (see Supplementary material 3). Notably, while 75% of older adults (or 82% if including the three older adults who could not complete the visual-only heading task) demonstrated lower visual-only JNDs post-training, only 64% of younger adults demonstrated lower visual-only JNDs post-training.

#### Bias values

**FIGURE 7:**
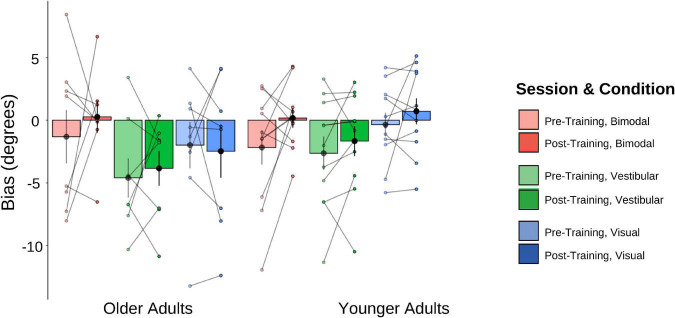
Biases plotted for each Age Group and sensory condition. Pre-training values (lighter shades) and post-training (darker shades). Individual data points are also plotted, with lines connecting each participant’s pre-training bias to their post-training bias, for each of the three sensory conditions (visual-only, vestibular-only, bimodal). Black dots represent means, plotted with standard error bars.

The pre-and post-training bias values are shown in Figure 7. A 2 (Age Group) × 3 (Condition) × 2 (Session) mixed-factorial ANOVA on perceptual biases showed a significant main effect of Condition [*F*(1.44, 24.41) = 5.58, *p* = 0.017], with *post-hoc t*-tests revealing significantly larger biases for the bimodal condition relative to the vestibular only condition [*t*(40) = −3.324, *p* = 0.011], but no significant differences for the bimodal condition relative to the visual-only condition, or the bimodal condition compared to the vestibular-only condition (*p*’s both > 0.05). There were no other significant main effects or interactions (i.e., no other effects of training).

#### Bias: Absolute difference scores

**FIGURE 8:**
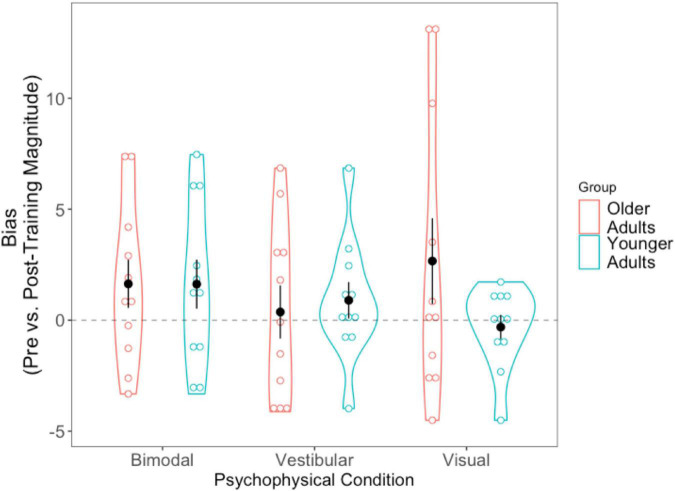
Difference scores for bias, across all three psychophysical conditions, for each of the two age groups. Black dots represent means, and error bars represent standard error. Individual data points are represented by the blue (younger adults) and red (older adults) circles. Black dashed line indicates a bias no closer to true straight ahead following training.

Absolute difference scores (pre-post) were calculated for the biases in the same way that they were for the JNDs. Getting closer to true straight ahead after training would result in a positive score. The 2 (Age Group) × 3 (Condition Difference Score) mixed-factorial ANOVA on perceptual biases did not show any significant main effects or interactions. We then conducted three separate pairwise *t*-tests to compare each condition’s difference bias scores (visual, vestibular, bimodal) with “0,” in order to assess whether changes in pre- and post-training biases differed significantly from zero. The results (Figure 8) showed that only the bimodal bias was significantly greater than 0 (i.e., closer to true straight ahead) following training, [*t*(21) = −2.149, *p* = 0.043].

As with the JNDs, we tallied the number of participants who demonstrated numeric post-training improvements in bias and report them for each sensory condition in Supplementary material 3.

### Maximum likelihood estimation

We calculated predicted optimal JNDs for both older and younger adults using an MLE model:


(1) $$J\,N\,D_{B\,i\,m\,o\,d\,a\,l}=\sqrt{\frac{J\,N\,D_{V\,i\,s\,u\,a\,l}^{2}+J\,N\,D_{V\,e\,s\,t\,i\,b\,u\,l\,a\,r}^{2}}{J\,N\,D_{V\,i\,s\,u\,a\,l}^{2}\times J\,N\,D_{V\,e\,s\,t\,i\,b\,u\,l\,a\,r}^{2}}}\,\ldots \,\ldots \,\ldots$$

**FIGURE 9:**
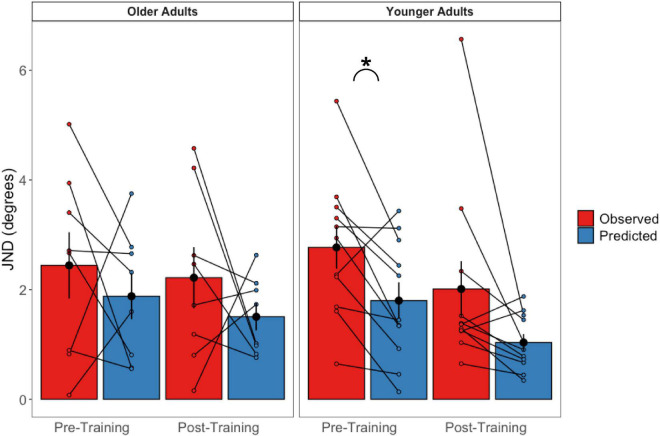
Observed bimodal JNDs relative to predicted bimodal JNDs in older (left panel) and younger (right panel) adults. Black dots represent the averages and error bars represent standard errors. Colored dots represent individual participant’s scores. *p < 0.05.

These were calculated for the pre-training and post-training bimodal session values separately (Figure 9). Paired sample *t*-tests revealed that older adults’ predicted JNDs did not differ significantly from their observed JNDs for either the pre-training session [*t*(7) = 0.776, *p* = 0.463] or the post-training session [*t*(7) = −1.855 *p* = 0.986]. For the younger adults, while predicted JNDs were significantly smaller (i.e., more precise) than their observed JNDs for the pre-training session [*t*(10) = −2.97, *p* = 0.014], we did not observe a significant difference in the post-training session [*t*(10) = −2.08 *p* = 0.064].

#### Older adults who were unable to estimate visual heading pre-training

Importantly, three of our older adult participants were not able to judge their heading in the visual-only condition before training and provided “left” and “right” responses essentially randomly during the task, despite understanding the instructions. Thus, their left- and right-starting PESTs for the visual-only condition did not converge, and we could not obtain meaningful JND or bias values for these participants. As such, they were removed from the group-level analyses described above. They were, however, able to perform the visual-only heading task following the training, indicating a profound improvement as a result of training. We report their individual data here in Tables 2A,B. Further details of their performance can be found in Supplementary material 2.

**TABLE 2A:** JNDs for the three older adults who could not perform the visual-only heading task pre-training, as well as the group average and standard deviations for the rest of the older adult group.

JNDs						
	Pre-training			Post-training		
	Visual	Vestibular	Bimodal	Visual	Vestibular	Bimodal
Older Adult 1	–	2.14	0.48	36.16	0.93	0.97
Older Adult 2	–	1.68	0.25	25.10	0.37	3.69
Older Adult 3	–	5.85	1.20	3.54	3.80	1.29
Average of other older adults (*n* = 8)	10.14 (8.18)	2.65 (1.61)	1.95 (1.67)	5.79 (7.11)	2.24 (1.34)	2.15 (1.48)

JNDs are given in degrees. The averages of the other older adults’ data are presented in the last row along with the corresponding standard deviations.

**TABLE 2B:** Biases for the three older adults who could not perform the visual-only heading task pre-training, as well as the group average and standard deviations for the rest of the older adult group.

Biases						
	Pre-training			Post-training		
	Visual	Vestibular	Bimodal	Visual	Vestibular	Bimodal
Older Adult 1	–	−1.68	−1.44	1.24	−6.00	−4.89
Older Adult 2	–	−0.67	1.51	6.71	−4.47	−4.12
Older Adult 3	–	1.86	−0.94	−7.03	−1.03	−0.75
Average of other older adults (*n* = 8)	−1.97 (5.27)	−4.60 (4.34)	−1.32 (6.02)	−2.47 (6.02)	−3.84 (3.96)	0.27 (3.61)

Biases are given in degrees. The averages of the other older adults’ data are presented in the last row along with the corresponding standard deviations.

#### Training data

**FIGURE 10:**
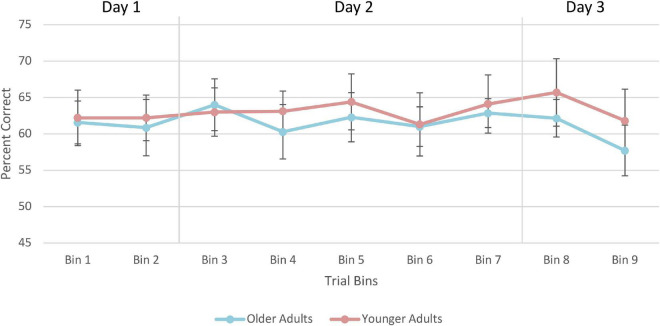
Average percent correct on the training trials for older and younger adults. Values for each bin represent the average percent correct for each 100 trials. Error bars represent standard error. Participants completed 250 trials on Day 1, 400 trials on Day 2, and 250 trials on Day 3. Older adult data are plotted in blue, and younger adult data in orange.

A 2 (Age Group) × 3 (Days) mixed-factorial ANOVA did not reveal any significant main effects of Days [*F*(1.41, 21.13) = 0.12, *p* = 0.82], Age Group [*F*(1, 15) = 0.11, *p* = 0.74], or interaction effects [*F*(1.41, 21.13) = 2.15, *p* = 0.15] (Figure 10).

#### Posturography task

**FIGURE 11:**
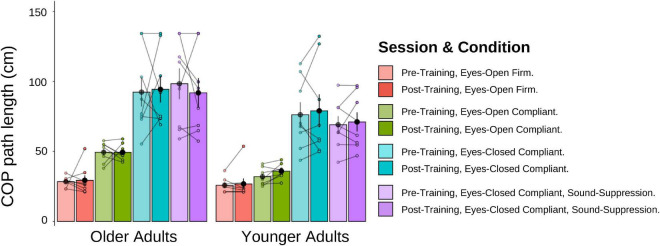
Mean COP path length (cm) for older and younger adults, pre-training (lighter shades) and post-training (darker shades). The individual data points are plotted for all four posturography conditions, with lines connecting each participant’s pre-training COP path length to their post-training COP path length. Black dots represent means, plotted with standard error bars.

A 2 (Age Group) × 4 (Posturography Condition) × 2 (Session) mixed-factorial ANOVA, with COP path length as the dependent variable was conducted (Figure 11). Data from the three older adults who could not estimate their visual pre-training heading were removed from these analyses. There was a main effect of Age Group [*F*(1,14) = 5.41, *p* = 0.036], indicating that older adults had significantly longer COP path lengths (*M* = 69.7 cm, *SD* = 42.3) than younger adults (*M* = 53.6 cm, *SD* = 30.2). There was also a main effect of Posturography Condition *F*(1.54, 21.50) = 48.87, *p* < 0.001], with pairwise comparisons showing that more difficult postural conditions produced significantly longer COP path lengths than each of the easier conditions (*p* < 0.05 between all conditions), with the exception of ECC compared to ECSS conditions (*p >* = 0.999). No other main effects or interactions were significant.

We conducted two additional mixed-factorial ANOVAs [2 (Age Group) × 4 (Posturography Condition) × 2 (Session)] with COP velocity and velocity-RMS as the dependent variables. Like the results of COP path length, older adults had larger velocity and velocity-RMS, compared to younger adults, with a similar main effect of Posturography Condition as described above, but no effect of training.

## Discussion

In this study, we examined whether older and younger adults would show reduced biases and/or smaller JNDs (i.e., increased precision) in their heading estimates following a visual-vestibular heading training task. Overall, we found a main effect of training for JND values (increased precision post-training relative to pre-training). Using difference scores, we observed that these training-related effects were only found in the visual-only condition and not in the bimodal or vestibular-only conditions. In line with previous studies investigating visual heading perception in older adults (Warren et al., 1989; Ramkhalawansingh et al., 2018) we found that three of our older adult participants (27% of our sample) were unable to perform the visual-only heading discrimination task in the pre-training phase of our study (Warren et al., 1989; Ramkhalawansingh et al., 2018). Importantly, however, all three of these participants were able to complete the visual-only heading task successfully after training (Table 2). We did not find improvements during the around-threshold training sessions, nor were there any changes in participants’ postural stability following training. Only the older (not younger) adults demonstrated a non-significant difference between predicted and obtained pre-training JNDs, which suggests optimal integration, in line with MLE model predictions. Both groups, however, showed optimal integration following training.

### Effects of training

Based on previous training studies (Hartmann et al., 2013; Fitzpatrick et al., 2015; Klaus et al., 2020; Diaz-Artiles and Karmali, 2021), we expected that we would observe improvements in heading perception following training. In support of these hypotheses, we did observe a main effect of training indicating that JNDs were reduced post-training compared to pre-training, with these training effects being mainly attributed to improvements in the vision-only condition. Following training, we also noted a profound improvement in the performance of three older adults who originally could not perform the visual-only task at all pre-training. This pronounced training effect for the visual-only condition could suggest that the sensory system with the poorest pre-training precision, in this case vision, benefited significantly from training with a bimodal input that included an additional, more precise sensory input (vestibular). While a unimodal training study would be needed to confirm this speculation, this interpretation is consistent with recent, mounting evidence demonstrating that multisensory training can facilitate perceptual improvements for unisensory tasks (Seitz et al., 2006; Von Kriegstein and Giraud, 2006; Shams and Seitz, 2008; Shams et al., 2011). Specifically, previous studies have shown that the benefits observed for unisensory tasks following multisensory training tend to exceed those obtained following unisensory training. For instance, in a recent audio-visual training study (Seitz et al., 2006), participants were provided with trial-by-trial feedback on a motion direction-detection task (i.e., “which of two intervals contained directional rather than random motion”). Participants who were trained on the audio-visual task demonstrated greater and faster improvements when tested on the visual-only motion task, relative to participants who were trained with only visual input. These results suggest that multisensory training might promote better learning for unisensory tasks than unisensory training alone.

Part of the reason we may have observed improvements during the post-training phase (relative to pre-training) but not during the training trials themselves may be because the training trials provided participants with subthreshold stimuli (i.e., ±67% of each participants’ bias). This method is consistent with previous multisensory training literature which found that sub-threshold, but not at-threshold or suprathreshold training, is associated with perceptual improvements.

While it is unclear why multisensory training may promote greater training benefits than unisensory training, it has been suggested that while unisensory training engages only primary sensory regions, multisensory learning engages several primary sensory regions (e.g., both auditory and visual cortices), as well as multisensory regions (e.g., parietal cortex), and functional as well as structural connections among these regions (Shams and Seitz, 2008). Such additional activation could account for some of the benefits observed from multisensory training, especially as it compares to unisensory training. In the present study, training would likely have recruited visual, vestibular, and bimodal regions (e.g., VIP, MSTd, insula; Fasold et al., 2002; Angelaki and Cullen, 2008; Lopez and Blanke, 2011) as well as connections among those regions. Furthermore, compared to training with only individual sensory inputs, multisensory inputs provide more information about a given object or event which could then be used to increase perceptual precision (Burr and Gori, 2011) and allow for calibration among the senses during training. This would be particularly beneficial for a sensory input that has a lower reliability when combined with a sensory input that has higher reliability, as was the case in the current study: visual-only heading perception was less precise than vestibular-only heading perception, especially for many of our older participants. In the present study, participants may have demonstrated greater post-training improvements in the visual-only condition (i.e., the least reliable pre-training condition), since this sensory condition was paired with redundant and congruent information from a more reliable sensory cue (i.e., vestibular) during training. Future studies could add a unisensory training condition (i.e., visual alone or vestibular alone) to examine whether multisensory training is indeed more effective than unisensory training in the visual-vestibular domain.

In the context of aging, very little is currently understood about whether older adults can benefit to the same or even greater extent from multisensory training as younger adults, given their often poorer overall precision and their heightened sensory integration (Ramkhalawansingh et al., 2018). This study is the first, to our knowledge, to show that self-motion perception, specifically in the context of visual-vestibular integration, can be improved following training. Interestingly, on average, the effects were not statistically different between age groups but most notable for three older adults who were initially completely unable to estimate their heading in the visual-only heading task pre-training, but were able to perform this task well post-training. Likewise, when examining difference scores, we found that participants showed improved (i.e., lower) JNDs for the visual-only condition relative to the bimodal after training, with results trending to suggest they were also lower than the JNDs in the vestibular condition (*p* = 0.057). The visual-only condition was also the only condition for which the training-related effects (difference scores) were significantly greater than zero.

### Transfer of training to standing balance stability

We also assessed the extent to which multisensory heading perception training might lead to changes in a standing balance task, given that both tasks rely on the precise integration of visual and vestibular cues. We did not, however, find any significant effects of bimodal heading training on balance performance. To our knowledge, there are no studies that have considered the effects of multisensory perceptual training on postural control. It has been shown, however, that poorer standing balance in older adults is associated with an increased susceptibility to the sound-induced flash illusion (Stapleton et al., 2014) and that training postural stability reduces susceptibility to such illusions (Merriman et al., 2015), suggesting that multisensory processing abilities may underlie both types of tasks.

One possible reason why effects of training did not transfer to postural control could be that our static posturography task may have been too easy and participants may have been able to take sufficient advantage of other, non-trained sensory inputs to successfully complete the tasks (e.g., proprioceptive/tactile). As such, using a more complex test of postural stability that would further challenge visual and/or vestibular abilities, such as dynamic posturography following a balance perturbation, might reveal some effects of visual-vestibular training (Baloh et al., 1998; Prosperini and Pozzilli, 2013).

## Conclusion

This study examined whether younger and older adults could be trained to better perceive self-motion (heading) after completing a multisensory training paradigm. We found that both younger and older adults became more precise in their visual-only performance following bimodal training. This improvement did not transfer to a static posturography task. Our results may have implications for mobility rehabilitation strategies, particularly in contexts when some sensory cues to self-motion are poor while others remain reliable.

## Data availability statement

The raw data supporting the conclusions of this article will be made available by the authors, without undue reservation.

## Ethics statement

The studies involving human participants were reviewed and approved by the University Health Network Research Ethics Board (Protocol Number: 18-5331.0) and University of Toronto Research Ethics Board (Protocol number: 00037394). The patients/participants provided their written informed consent to participate in this study.

## Author contributions

LH, DH, and JC conceived the study. GG, LH, DH, and JC helped design the study. GG and MP collected the data. GG, LH, and JC analyzed and interpreted the data. GG and JC drafted the manuscript. All authors contributed to critically revising the manuscript.

## Footnotes

1
https://www.stereooptical.com/
