# Supplementary material for: Multisensory visual-vestibular training improves visual heading estimation in younger and older adults
Source: Front Aging Neurosci. 2022 Aug 25;14:816512. doi: 10.3389/fnagi.2022.816512 (PMC9452741; doi:10.3389/fnagi.2022.816512)
Supplement: Supplementary file 1 [file Data_Sheet_1.docx]

**Supplemental Materials S.1**

In the following analyses, we repeat the analyses on the heading data in the current manuscript (ANOVAs) however, this time, we winsorize the data. Specifically, all data were winsorized to treat potential outliers using the “DescTools” R package (Signorell et al., 2019). With this winsorization function all values less than 5%-quantile, or greater than 5%+quantile, were replaced by 5%-quantile and 5%+quantile, respectively.

Otherwise, as with the main manuscript, two separate mixed-factorial ANOVAs, 2 (Age Group; younger, older) $\times$ 3 (Psychophysical Condition; visual-only, vestibular-only, bimodal) $\times$ 2 (Session; pre-training, post-training), were conducted to evaluate the extent to which participants’ perceptual biases and JNDs (dependent variables) changed in the older and younger groups following training, for each of the three psychophysical conditions (visual-only, vestibular-only, bimodal). Once again, post-hoc *t*-tests were Tukey-corrected for multiple comparisons.

**Heading**

**Precision.**

The pre-and post-training JND values are shown in Figure S.1.1. The 2 (Age Group) $\times$ 3 (Psychophysical Condition) $\times$ 2 (Session) mixed-factorial ANOVA on JND values revealed a main effect of Session [*F*(1, 17) = 9.16, *p* = .008], indicating that pre-training JNDs were significantly larger (i.e., worse) than post-training JNDs. It also showed a main effect of Condition [*F*(1, 17) = 8.34, *p* = .002], with post-hoc *t*-tests revealing that JNDs in the bimodal and vestibular-only conditions were significantly smaller than the JNDs in the visual-only condition ([*t*(17) = -1.19, *p* = .012] and [*t*(17) = -1.24, *p* = .010] respectively) (Figure S.1.1).

There were no other significant differences, which includes a trending Session $\times$ Condition interaction [*F*(1.62, 27.50) = 3.16, *p* = .068]. Post-hoc *t-*tests suggest that the visual-only condition may have been driving this interaction [*t*(17) = 2.74, *p* =.014].

*Figure S.1.1.* Mean and individual JND values plotted for each Age Group and sensory condition, pre-training (lighter shades) and post-training (darker shades). Data were winsorized before plotting and analysing. Individual data points are also plotted, with lines connecting each participant’s pre-training JND to their post-training JND for each of the three sensory conditions (visual-only, vestibular-only, bimodal). Black dots represent means, plotted with standard error bars.

**Bias.**

The pre-and post-training bias values are shown in Figure S.1.2. A 2 (Age Group) $\times$ 3 (Psychophysical Condition) $\times$ 2 (Session) mixed-factorial ANOVA on perceptual biases showed a significant main effect of Condition [*F*(1.43, 24.29) = 5.56, *p* = .017], with post-hoc *t*-tests revealing significantly larger biases for the bimodal condition relative to the vestibular only condition [*t*(17) = 3.319, *p* = .012], but no significant differences for the bimodal condition relative to the visual-only condition, or the bimodal condition compared to the vestibular-only condition (*p*’s both > 0.05). There were no other significant main effects or interactions.

*Figure S.1.2.* Perceptual biases plotted for each Age Group and sensory condition. Data were winsorized before plotting and analysing. Pre-training values (lighter shades) and post-training (darker shades). Individual data points are also plotted, with lines connecting each participant’s pre-training bias to their post-training bias, for each of the three sensory conditions (visual-only, vestibular-only, bimodal). Black dots represent means, plotted with standard error bars.

**Supplemental Materials S.2**

**Three Older Adults**

Three older adult participants were unable to produce data that could be reliably fit to a psychometric function during the pre-training visual-condition. We attach their data below.

# Participant #1

## **Pre-Training Data**

Visual Condition: Bias: -11.00446; JND: 20.00000 r^2^: 0.03162

***Figure S.2.1* a)** The two interleaved PEST staircases in the top panel with the right-starting PEST is plotted in purple and the left-starting PEST in blue. Open blue circles represent each trial. 0$^{\circ}$ represents straight-ahead, with positive values representing rightward angles, and negative values leftward angles. The dotted lines represent the bias obtained for each individual PEST. **b)** In the bottom panel, open blue circles represent the participant’s response to each trial (1 = right, -1 = left) for each presented heading angle. The solid red line is the fitted logistic function. The dotted red line represents the midpoint of this function (i.e., participant’s perceptual bias).

Bimodal Condition: Bias: -1.44217; JND: 0.47776; r^2^: 0.33241

***Figure S.2.2* a)** The two interleaved PEST staircases in the top panel with the right-starting PEST is plotted in purple and the left-starting PEST in blue. Open blue circles represent each trial. 0$^{\circ}$ represents straight-ahead, with positive values representing rightward angles, and negative values leftward angles. The dotted lines represent the bias obtained for each individual PEST. **b)** In the bottom panel, open blue circles represent the participant’s response to each trial (1 = right, -1 = left) for each presented heading angle. The solid red line is the fitted logistic function. The dotted red line represents the midpoint of this function (i.e., participant’s perceptual bias).

Vestibular Condition: Bias: -1.68226; JND: 2.14382; r^2^: 0.31540

***Figure S.2.3* a)** The two interleaved PEST staircases in the top panel with the right-starting PEST is plotted in purple and the left-starting PEST in blue. Open blue circles represent each trial. 0$^{\circ}$ represents straight-ahead, with positive values representing rightward angles, and negative values leftward angles. The dotted lines represent the bias obtained for each individual PEST. **b)** In the bottom panel, open blue circles represent the participant’s response to each trial (1 = right, -1 = left) for each presented heading angle. The solid red line is the fitted logistic function. The dotted red line represents the midpoint of this function (i.e., participant’s perceptual bias).

## **Post-Training Data**

Visual-Only Condition: Bias: 1.23840; JND: 20.00000; r^2^: 0.04115

***Figure S.2.4* a)** The two interleaved PEST staircases in the top panel with the right-starting PEST is plotted in purple and the left-starting PEST in blue. Open blue circles represent each trial. 0$^{\circ}$ represents straight-ahead, with positive values representing rightward angles, and negative values leftward angles. The dotted lines represent the bias obtained for each individual PEST. **b)** In the bottom panel, open blue circles represent the participant’s response to each trial (1 = right, -1 = left) for each presented heading angle. The solid red line is the fitted logistic function. The dotted red line represents the midpoint of this function (i.e., participant’s perceptual bias).

Bimodal Condition: Bias: -4.89313; JND: 0.97028; r^2^: 0.35648 ***Figure S.2.5* a)** The two interleaved PEST staircases in the top panel with the right-starting PEST is plotted in purple and the left-starting PEST in blue. Open blue circles represent each trial. 0$^{\circ}$ represents straight-ahead, with positive values representing rightward angles, and negative values leftward angles. The dotted lines represent the bias obtained for each individual PEST. **b)** In the bottom panel, open blue circles represent the participant’s response to each trial (1 = right, -1 = left) for each presented heading angle. The solid red line is the fitted logistic function. The dotted red line represents the midpoint of this function (i.e., participant’s perceptual bias).

Vestibular Condition: Bias: -5.99564; JND: 0.93273; r^2^: 0.33198 ***Figure S.2.6* a)** The two interleaved PEST staircases in the top panel with the right-starting PEST is plotted in purple and the left-starting PEST in blue. Open blue circles represent each trial. 0$^{\circ}$ represents straight-ahead, with positive values representing rightward angles, and negative values leftward angles. The dotted lines represent the bias obtained for each individual PEST. **b)** In the bottom panel, open blue circles represent the participant’s response to each trial (1 = right, -1 = left) for each presented heading angle. The solid red line is the fitted logistic function. The dotted red line represents the midpoint of this function (i.e., participant’s perceptual bias).

# Participant #2

## **Pre-Training Data**

Visual-Only Condition: Bias: -20.00000; JND: 20.00000; r^2^: -1.20263

 ***Figure S.2.7* a)** The two interleaved PEST staircases in the top panel with the right-starting PEST is plotted in purple and the left-starting PEST in blue. Open blue circles represent each trial. 0$^{\circ}$ represents straight-ahead, with positive values representing rightward angles, and negative values leftward angles. The dotted lines represent the bias obtained for each individual PEST. **b)** In the bottom panel, open blue circles represent the participant’s response to each trial (1 = right, -1 = left) for each presented heading angle. The solid red line is the fitted logistic function. The dotted red line represents the midpoint of this function (i.e., participant’s perceptual bias).

Bimodal Condition: Bias: 1.51090; JND: 0.25308; r^2^: 0.39968

 ***Figure S.2.8* a)** The two interleaved PEST staircases in the top panel with the right-starting PEST is plotted in purple and the left-starting PEST in blue. Open blue circles represent each trial. 0$^{\circ}$ represents straight-ahead, with positive values representing rightward angles, and negative values leftward angles. The dotted lines represent the bias obtained for each individual PEST. **b)** In the bottom panel, open blue circles represent the participant’s response to each trial (1 = right, -1 = left) for each presented heading angle. The solid red line is the fitted logistic function. The dotted red line represents the midpoint of this function (i.e., participant’s perceptual bias).

Vestibular Condition: Bias: -0.66607; JND: 1.67692; r^2^: 0.35302 ***Figure S.2.9* a)** The two interleaved PEST staircases in the top panel with the right-starting PEST is plotted in purple and the left-starting PEST in blue. Open blue circles represent each trial. 0$^{\circ}$ represents straight-ahead, with positive values representing rightward angles, and negative values leftward angles. The dotted lines represent the bias obtained for each individual PEST. **b)** In the bottom panel, open blue circles represent the participant’s response to each trial (1 = right, -1 = left) for each presented heading angle. The solid red line is the fitted logistic function. The dotted red line represents the midpoint of this function (i.e., participant’s perceptual bias).

## **Post-Training Data**

Visual Condition: Bias: 6.71115 JND: 20.00000 r^2^: 0.10642

 ***Figure S.2.10* a)** The two interleaved PEST staircases in the top panel with the right-starting PEST is plotted in purple and the left-starting PEST in blue. Open blue circles represent each trial. 0$^{\circ}$ represents straight-ahead, with positive values representing rightward angles, and negative values leftward angles. The dotted lines represent the bias obtained for each individual PEST. **b)** In the bottom panel, open blue circles represent the participant’s response to each trial (1 = right, -1 = left) for each presented heading angle. The solid red line is the fitted logistic function. The dotted red line represents the midpoint of this function (i.e., participant’s perceptual bias).

Bimodal Condition: Bias: -4.12333; JND: 3.69488; r^2^: 0.22434 ***Figure S.2.11* a)** The two interleaved PEST staircases in the top panel with the right-starting PEST is plotted in purple and the left-starting PEST in blue. Open blue circles represent each trial. 0$^{\circ}$ represents straight-ahead, with positive values representing rightward angles, and negative values leftward angles. The dotted lines represent the bias obtained for each individual PEST. **b)** In the bottom panel, open blue circles represent the participant’s response to each trial (1 = right, -1 = left) for each presented heading angle. The solid red line is the fitted logistic function. The dotted red line represents the midpoint of this function (i.e., participant’s perceptual bias).

Vestibular-Only Condition: Bias: -4.47834; JND: 0.36539; r^2^: 0.33997

 ***Figure S.2.12* a)** The two interleaved PEST staircases in the top panel with the right-starting PEST is plotted in purple and the left-starting PEST in blue. Open blue circles represent each trial. 0$^{\circ}$ represents straight-ahead, with positive values representing rightward angles, and negative values leftward angles. The dotted lines represent the bias obtained for each individual PEST. **b)** In the bottom panel, open blue circles represent the participant’s response to each trial (1 = right, -1 = left) for each presented heading angle. The solid red line is the fitted logistic function. The dotted red line represents the midpoint of this function (i.e., participant’s perceptual bias).

# Participant #3

## **Pre-Training Data**

Visual-Only Data: Bias: 20.00000; JND: 20.00000; r^2^: -1.33218 ***Figure S.2.13* a)** The two interleaved PEST staircases in the top panel with the right-starting PEST is plotted in purple and the left-starting PEST in blue. Open blue circles represent each trial. 0$^{\circ}$ represents straight-ahead, with positive values representing rightward angles, and negative values leftward angles. The dotted lines represent the bias obtained for each individual PEST. **b)** In the bottom panel, open blue circles represent the participant’s response to each trial (1 = right, -1 = left) for each presented heading angle. The solid red line is the fitted logistic function. The dotted red line represents the midpoint of this function (i.e., participant’s perceptual bias).

Bimodal Condition: Bias: -0.94336; JND: 1.20034; r^2^: 0.38696 ***Figure S.2.14* a)** The two interleaved PEST staircases in the top panel with the right-starting PEST is plotted in purple and the left-starting PEST in blue. Open blue circles represent each trial. 0$^{\circ}$ represents straight-ahead, with positive values representing rightward angles, and negative values leftward angles. The dotted lines represent the bias obtained for each individual PEST. **b)** In the bottom panel, open blue circles represent the participant’s response to each trial (1 = right, -1 = left) for each presented heading angle. The solid red line is the fitted logistic function. The dotted red line represents the midpoint of this function (i.e., participant’s perceptual bias).

Vestibular Condition: Bias: 1.85911; JND: 5.85284; r^2^: 0.28093 ***Figure S.2.15* a)** The two interleaved PEST staircases in the top panel with the right-starting PEST is plotted in purple and the left-starting PEST in blue. Open blue circles represent each trial. 0$^{\circ}$ represents straight-ahead, with positive values representing rightward angles, and negative values leftward angles. The dotted lines represent the bias obtained for each individual PEST. **b)** In the bottom panel, open blue circles represent the participant’s response to each trial (1 = right, -1 = left) for each presented heading angle. The solid red line is the fitted logistic function. The dotted red line represents the midpoint of this function (i.e., participant’s perceptual bias).

## **Post-Training Data**

Visual-Only Condition: Bias: 5.03580; JND: 3.54377; r^2^: 0.12758

 ***Figure S.2.16* a)** The two interleaved PEST staircases in the top panel with the right-starting PEST is plotted in purple and the left-starting PEST in blue. Open blue circles represent each trial. 0$^{\circ}$ represents straight-ahead, with positive values representing rightward angles, and negative values leftward angles. The dotted lines represent the bias obtained for each individual PEST. **b)** In the bottom panel, open blue circles represent the participant’s response to each trial (1 = right, -1 = left) for each presented heading angle. The solid red line is the fitted logistic function. The dotted red line represents the midpoint of this function (i.e., participant’s perceptual bias).

Bimodal Condition: Bias: 1.18559; JND: 1.28769; r^2^: 0.28192 ***Figure S.2.17* a)** The two interleaved PEST staircases in the top panel with the right-starting PEST is plotted in purple and the left-starting PEST in blue. Open blue circles represent each trial. 0$^{\circ}$ represents straight-ahead, with positive values representing rightward angles, and negative values leftward angles. The dotted lines represent the bias obtained for each individual PEST. **b)** In the bottom panel, open blue circles represent the participant’s response to each trial (1 = right, -1 = left) for each presented heading angle. The solid red line is the fitted logistic function. The dotted red line represents the midpoint of this function (i.e., participant’s perceptual bias).

Vestibular-Only Condition: Bias: 1.94416; JND: 3.80622; r^2^: 0.27610 ***Figure S.2.18* a)** The two interleaved PEST staircases in the top panel with the right-starting PEST is plotted in purple and the left-starting PEST in blue. Open blue circles represent each trial. 0$^{\circ}$ represents straight-ahead, with positive values representing rightward angles, and negative values leftward angles. The dotted lines represent the bias obtained for each individual PEST. **b)** In the bottom panel, open blue circles represent the participant’s response to each trial (1 = right, -1 = left) for each presented heading angle. The solid red line is the fitted logistic function. The dotted red line represents the midpoint of this function (i.e., participant’s perceptual bias).

**Supplemental Materials S.3**

# Numeric Improvements After Training

Here we calculated the sum of participants in each group (older and younger adults) who demonstrated numeric post-training improvements (Table S.3.1) – specifically numerically smaller biases and smaller JNDs after training.

Table S.3.1 Numeric Post-Training Improvement

|  | Visual  Bias | Visual  JND | Bimodal Bias | Bimodal JND | Vestibular Bias | Vestibular JND |
| --- | --- | --- | --- | --- | --- | --- |
| **Younger Adult Improvement** |  |  |  |  |  |  |
| *n /* total | 6 / 11 | 7 / 11 | 7 / 11 | 9 / 11 | 7 / 11 | 5 / 11 |
| % | 55% | 64% | 64% | 82% | 64% | 45% |
|  |  |  |  |  |  |  |
| **Older Adult Improvement** |  |  |  |  |  |  |
| *n /* total | 3 / 8* | 6 / 8* | 7 / 11 | 5 / 11 | 5 / 11 | 7 / 11 |
| % | 38%* | 75%* | 64% | 45% | 45% | 64% |

Note: *The three older adults who could not perform in the pre-training visual task were removed from the visual task improvement scores listed in the table. If they are added, then 9/11 (82%) older adults demonstrated visual JND improvements and 6/11 (55%) showed visual bias improvements.

**Supplemental Materials S.4**

Predicted vs. Observed Weights After Training using a Maximum Likelihood Estimation (MLE) model

We compared observed visual weights ($\frac{{Bias}_{Bimodal}-{Bias}_{Visual}}{{Bias}_{Vestibular}-{Bias}_{Visual}}$) and observed vestibular weights ($\frac{{Bias}_{Vestibular}- {Bias}_{Bimodal}}{{Bias}_{Vestibular}-{Bias}_{Visual}}$,) to predicted visual and vestibular weights, respectively (obtained by $\frac{{{JND}_{Vestibular}}^{2}}{{{JND}_{Vestibular}}^{2}+ {{JND}_{Visual}}^{2}}$ and $\frac{{{JND}_{Visual}}^{2}}{{{JND}_{Vestibular}}^{2}+ {{JND}_{Visual}}^{2}}$, respectively). Specifically, we conducted a series of two-tailed, paired-sample *t-*tests, for older and younger adults separately. The results did not reveal a significant difference between observed and predicted weights for the visual condition, nor for the vestibular condition in older adults (Figure S.4.1) (*p_Pre-Training_* = .26 and *p_Post-Training_* = .54), or younger adults (Figure S.4.2) (*p_Pre-Training_* = .11 and *p_Post-Training_* = .73). We also conducted a separate ANOVA (Age Group $\times$Sensory Condition $\times$Training Session $\times$ Observed/Predicted) and found no significant main effects or interactions.


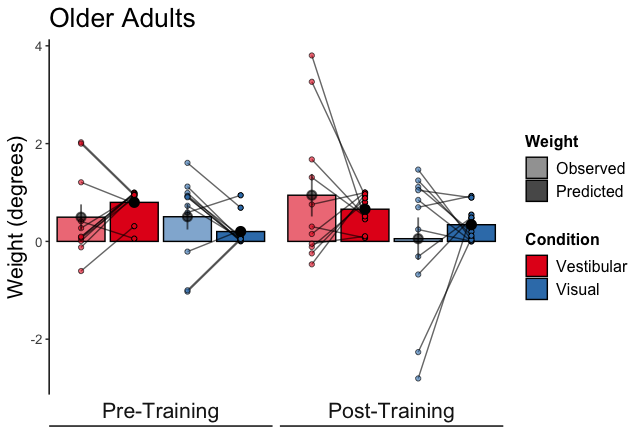


*Figure S.4.1.* Graph representing observed (lighter shade) and predicted (darker shade) sensory weightings for the visual (blue) and vestibular (red) conditions. Data from only the older adult participants are represented. Individual data points are plotted, as are means (black circles) and standard error bars.


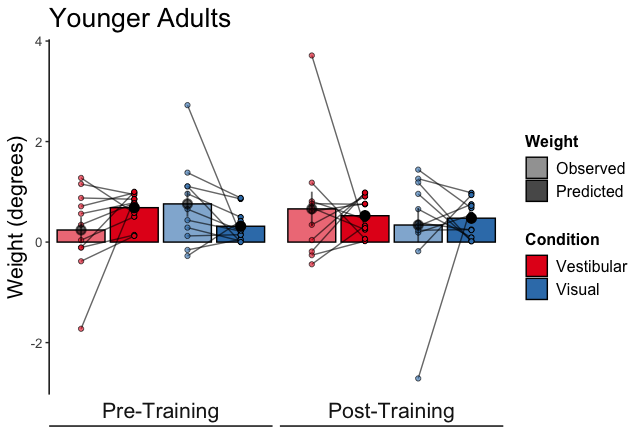


*Figure S.4.2.* Graph representing observed (lighter shade) and predicted (darker shade) sensory weightings for the visual (blue) and vestibular (red) conditions. Data from only the younger adult participants are represented. Individual data points are plotted, as are means (black circles) and standard error bars.
